# A Complex Systems Approach to Evaluate HIV Prevention in Metropolitan Areas: Preliminary Implications for Combination Intervention Strategies

**DOI:** 10.1371/journal.pone.0044833

**Published:** 2012-09-13

**Authors:** Brandon D. L. Marshall, Magdalena M. Paczkowski, Lars Seemann, Barbara Tempalski, Enrique R. Pouget, Sandro Galea, Samuel R. Friedman

**Affiliations:** 1 Department of Epidemiology, Columbia University Mailman School of Public Health, New York, New York, United States of America; 2 Department of Epidemiology, Brown University, Providence, Rhode Island, United States of America; 3 Department of Physics, University of Houston, Houston, Texas, United States of America; 4 Institute for AIDS Research, National Development and Research Institutes, Inc., New York, New York, United States of America; Yale School of Public Health, United States of America

## Abstract

**Background:**

HIV transmission among injecting and non-injecting drug users (IDU, NIDU) is a significant public health problem. Continuing propagation in endemic settings and emerging regional outbreaks have indicated the need for comprehensive and coordinated HIV prevention. We describe the development of a conceptual framework and calibration of an agent-based model (ABM) to examine how combinations of interventions may reduce and potentially eliminate HIV transmission among drug-using populations.

**Methodology/Principal Findings:**

A multidisciplinary team of researchers from epidemiology, sociology, geography, and mathematics developed a conceptual framework based on prior ethnographic and epidemiologic research. An ABM was constructed and calibrated through an iterative design and verification process. In the model, “agents” represent IDU, NIDU, and non-drug users who interact with each other and within risk networks, engaging in sexual and, for IDUs, injection-related risk behavior over time. Agents also interact with simulated HIV prevention interventions (e.g., syringe exchange programs, substance abuse treatment, HIV testing) and initiate antiretroviral treatment (ART) in a stochastic manner. The model was constructed to represent the New York metropolitan statistical area (MSA) population, and calibrated by comparing output trajectories for various outcomes (e.g., IDU/NIDU prevalence, HIV prevalence and incidence) against previously validated MSA-level data. The model closely approximated HIV trajectories in IDU and NIDU observed in New York City between 1992 and 2002, including a linear decrease in HIV prevalence among IDUs. Exploratory results are consistent with empirical studies demonstrating that the effectiveness of a combination of interventions, including syringe exchange expansion and ART provision, dramatically reduced HIV prevalence among IDUs during this time period.

**Conclusions/Significance:**

Complex systems models of adaptive HIV transmission dynamics can be used to identify potential collective benefits of hypothetical combination prevention interventions. Future work will seek to inform novel strategies that may lead to more effective and equitable HIV prevention strategies for drug-using populations.

## Introduction

Although the global incidence of HIV infection has declined by over 20% since its peak in 1997, the persistent large number of new infections annually, estimated at 2.6 million in 2009 [1], demonstrates that the need for effective HIV prevention strategies remains urgent. A series of recently published efficacious interventions [2], [3], [4] have renewed interest in placing prevention-centered approaches at the center of global HIV elimination strategies. However, as it is increasing likely that no single biomedical intervention will be sufficient to control HIV/AIDS and also that social and behavioral aspects of implementing such biomedical interventions need to be considered [5], [6], there is an emerging consensus that combination HIV prevention (i.e., packages of evidence-based interventions tailored to specific populations) provides the best opportunity to significantly reduce HIV transmission at the population level [7], [8], [9]. In this paper, we will adopt the term “highly active HIV prevention” to refer to the additive (and potentially interactive) effect of combining treatment-centered approaches, biomedical strategies, behavioral interventions, and structural changes to suppress and eventually eliminate HIV transmission [10].

**Table 1:** Initial population distribution of the agent-based model (row percentages).

Population Group	Male		Female		Total
	MSM	HM	WSW	HF	
IDU	7.0%	63.0%	5.1%	24.9%	1.9%
NIDU	7.8%	57.2%	6.0%	29.0%	6.4%
NU	2.4%	45.3%	1.7%	50.6%	91.7%
**Total**	3.0%	47.0%	2.5%	47.5%	100.0%

Abbreviations: HF – heterosexual female; HM – heterosexual male; IDU – injection drug user; MSM -men who have sex with men; NIDU – non-injection drug user; NU – non-drug user; WSW – women who have sex with women.

Note: proportions estimated empirically from: [68], [69], [71], [72], [73], [74], [75], [76], [77], [78], [79], [80], [107], [163].

Although the Joint United Nations Programme on HIV/AIDS (UNAIDS) has formally adopted combination prevention as a key component in a new global HIV strategy and has recommended that these programs be expanded immediately [11], epidemiologic evidence to guide the implementation of “highly active” HIV prevention continues to be scarce. Given that adverse effects may arise from combining interventions that have been found to be efficacious in individual randomized controlled trials or from the inclusion of unproven interventions within combination prevention packages [12], there is a need to investigate which sets of programs produce maximal sustained benefit under limited resource allocation scenarios [13], [14].

In this paper we examine how complex systems approaches can contribute to the evolving field of HIV epidemiology and prevention. As an illustration of these techniques, we then describe the construction and calibration of an agent-based model (ABM) of HIV transmission within a population-based sexual and injecting network. ABMs are computational models used to simulate autonomous “agents” (i.e., individuals) interacting within a shared environment. ABMs can and have been used to examine how multi-level policies and programs shape population health [15].

**Table 2:** Key parameter values for injection drug-using (IDU) agents

Parameter	MSM	HM	HF	WSW	Sources
**Baseline Prevalence (%)**					
HIV	55.0	42.0	39.0	53.0	_ENREF_14CVAR, [47], [102]
AIDS	5.8†				CVAR
**Mortality Rate (per 1,000 person-years)**					
HIV negative	15				[164], [165]
HIV positive, not on HAART	100				[166]
HIV positive, on HAART	23				[63], [167]
AIDS	200				[168], [169]
**Progression to AIDS (annual probability)**					
Not on HAART	0.167				[143], [144]
On HAART and ≥90% adherent to therapy	0.067				[63]
On HAART and <90% adherent to therapy	See Table 5				
**Number of contacts (per time step)**	Poisson (λ = 5)				[88], [89], [90]
**Sexual Behavior (annual probability)**					
Unprotected intercourse‡	0.75	0.75	0.75	0.75	[83], [102], [103], [104]
Unprotected intercourse‡ at *t = j*, given VCT = + at *t* < j*	0.65	0.45	0.45	0.45	[83], [111], [112], [113]
**Injecting Behavior (annual probability)**					
Consistent NSP use¶, given *t* <1995, no treatment	0.65				[47], [116]
Consistent NSP use ¶, given *t* <1995, treatment	0.85				[66], [170]
Consistent NSP use ¶, given *t* ≥1995, no treatment	0.80				[44]
Consistent NSP use ¶, given *t* ≥1995, treatment	0.90				[66], [170]
**Substance Abuse Treatment (annual probability)**					
Treatment initiation at *t = j*, given no NSP use at *t = j*	0.09				[53], [118]
Treatment initiation at *t = j*, given NSP use at *t = j*	0.18				[53], [55], [56]
Discontinuation§ at *t = j*, given initiation at *t* < *j*	0.50				[53], [119]
**Voluntary HIV Testing (annual probability)**					
Access VCT at *t = j*, given no NSP use at *t = j*	0.25				[54]
Access VCT at *t = j*, given NSP use at *t = j*	0.45				[54]
**HIV Treatment (annual probability)**					
HAART initiation, given *t* ≤1996	0.00				[171]
HAART initiation, given *t* >1996, no treatment	0.08				[65]
HAART initiation, given *t* >1996, treatment	0.14				[65], [172]
Discontinuation at *t* = *j*, given initiation at *t*<*j*	0.45				[65], [139], [140], [141]

Abbreviations: AIDS – acquired immune deficiency syndrome; HAART – highly active antiretroviral therapy; HIV – human immunodeficiency virus; HF – heterosexual female; HM – heterosexual male; MSM – men who have sex with men; NSP – needle and syringe exchange program; VCT – voluntary counseling and HIV testing; WSW – women who have sex with women.

Notes:

† AIDS prevalence within the entire IDU population;

‡ <100% correct condom use between agent dyads; * defined as accessing VCT at *t* < *j* and testing positive for HIV;

¶ synonymous with 100% sterile syringe use;

§ agents who discontinue treatment at *t = j* can re-initiate treatment at some *t* > *j* with probability *P* = 0.18.

Although many different combinations of biomedical, behavioral, policy, and structural interventions can be integrated into the model and will be examined in future work, we will focus this paper on four evidence-based approaches (i.e., needle and syringe programs [NSPs], substance abuse treatment, voluntary counseling and HIV testing [VCT], provision of highly active antiretroviral therapy [HAART]) used to prevent HIV transmission among injection and non-injection drug users (IDU, NIDU). As such, a brief overview of the epidemiology of drug use and HIV is provided for readers. We conclude with a discussion of the benefits and challenges of incorporating complex systems methods within epidemiology and HIV prevention science.

**Table 3:** Key parameter values and sources for non-injection drug-using (NIDU) agents.

Parameter	MSM		HM	HF	WSW	Sources
**Baseline Prevalence (%)**						
HIV	18.0		4.8	4.8	4.8	CVAR, [40], [79], [81], [95]
AIDS	2.0†	0.2†				CVAR, [173]
**Mortality Rate (per 1,000 person-years)**						
HIV negative	7					[165]
HIV positive, not on HAART	25					[173]
HIV positive, on HAART	18					[173]
AIDS	80					[173]
**Progression to AIDS (annual probability)**						
Not on HAART	0.100					[143], [144]
On HAART and ≥90% adherent to therapy	0.010					[63]
On HAART and <90% adherent to therapy	See Table 5					
**Number of contacts (per time step)**	Poisson (λ = 3)					[91], [92], [93]
**Sexual Behavior (annual probability)**						
Unprotected intercourse‡	0.40		0.70	0.70	0.75	[78], [80], [106], [107]
Unprotected intercourse‡ at *t = j*, given VCT = + at *t* < *j* *	0.40		0.35	0.35	0.45	[60], [61], [115]
**Voluntary HIV Testing (annual probability)**						
Access VCT	0.25	0.06				[82], [174]
**HIV Treatment (annual probability)**						
HAART initiation, given *t* ≤1996	0.00					[171]
HAART initiation, given *t* >1996	0.14					[175]
Discontinuation at *t* = *j*, given initiation at *t*<*j*	0.35					[141], [142]

Abbreviations: AIDS – acquired immune deficiency syndrome; HAART – highly active antiretroviral therapy; HIV – human immunodeficiency virus; HF – heterosexual female; HM – heterosexual male; MSM – men who have sex with men; NSP – needle and syringe exchange program; VCT – voluntary counseling and HIV testing; WSW – women who have sex with women.

Notes:

† value represents the prevalence of AIDS within the entire population of NIDU;

‡ defined as <100% correct condom use between agent dyads;

* defined as accessing VCT at *t* < *j* and testing positive for HIV.

### Complex Systems Approaches in Epidemiology

The use of traditional epidemiologic studies to assess the effectiveness of combination HIV prevention strategies is limited by several factors, including methodological challenges, ethical considerations, cost, and the scale necessary to observe intended effects [16]. In order to identify both the independent and synergistic effects of multiple interventions, very large studies using complicated factorial designs are required [17]. For example, even with only four interventions, 2^4^ = 16 randomized blocks would be required. Furthermore, traditional epidemiologic approaches, including those relying on regression analyses, seek to identify the independent risk factors for a specific health outcome, and are thus often unable to account for the interdependent, non-linear, and adaptive processes that occur as individuals interact with each other and their environments [18]. Under these research paradigms, important systems-level processes, including interactive feedback loops among system components and across levels of analysis, social learning in networks, and individual-level reciprocity may go unrecognized [19]. In the absence of models that account for these dynamics, interventions (including for example programs that target one exposure or risk factor in the absence of others) may inadvertently increase health disparities and many even lead to outbreaks in vulnerable sub-populations [20].

**Table 4:** Key parameter values and sources for agents who do not use drugs (NU).

Parameter	MSM		HM	HF	WSW	Sources
**Baseline Prevalence (%)**						
HIV	8.0		1.5	1.2	1.2	[69], [81], [176]
AIDS	2.0†	0.03†				CVAR
**Mortality Rate (per 1,000 person-years)**						
HIV negative	5					[177]
HIV positive, not on HAART	40					[178]
HIV positive, on HAART	10					[62], [63]
AIDS	80					[144]
**Progression to AIDS (annual probability)**						
Not on HAART	0.100					[143], [144]
On HAART and ≥90% adherent to therapy	0.010					[63]
On HAART and <90% adherent to therapy	See Table 5					
**Number of Contacts (per time step)**	Poisson (λ = 1.5, 1.0)§					[73], [82], [97], [98], [99]
**Sexual Behavior (annual probability)**						
Unprotected intercourse‡	0.40		0.70	0.70	0.75	[101], [108], [109], [110]
Unprotected intercourse‡ at *t = j*, given VCT = + at *t* < *j* *	0.40		0.35	0.35	0.45	[60], [61], [115]
**Voluntary HIV Testing (annual probability)**						
Access VCT	0.25	0.06				[82], [174]
**HIV Treatment (annual probability)**						
HAART initiation, given *t* ≤1996	0.00					[171]
HAART initiation, given *t* >1996	0.14					[179]
Discontinuation at *t* = *j*, given initiation at *t*<*j*	0.35					[141], [142]

Abbreviations: AIDS – acquired immune deficiency syndrome; HAART – highly active antiretroviral therapy; HIV – human immunodeficiency virus;

HF – heterosexual female; HM – heterosexual male; MSM – men who have sex with men; VCT – voluntary counseling and HIV testing; WSW – women who have sex with women.

Notes:

† value represents the prevalence of AIDS within the entire population of NU;

‡ defined as <100% correct condom use between agent dyads;

* defined as accessing VCT at *t* < *j* and testing positive for HIV;

§ MSM and WSW partners sampled from a Poisson distribution with mean 1.5, HM and HF are sampled from a Poisson distribution with mean 1.0.

In contrast, complex systems methods rely on computer algorithms to model dynamic and evolving interactions among individuals and their environments [21]. They permit the researcher to study the impact of particular perturbations (including hypothetical interventions) on population health in simulated environments [22]. The methods can be used to integrate empiric data from a large number of studies and contexts, which permits the simulation of interventions within various population structures with greater efficiency than the replication of observational studies and trials. Given these advantages, it is not surprising that complex systems approaches have been used extensively to model a wide variety of health behaviors and other social phenomena [23], [24], [25]. Furthermore, calls to integrate complex systems approaches within public health science are increasingly common [22], [26], [27], [28]. Although these methods are gaining traction in epidemiology, their practical utility to address “real-world” public health problems largely remains to be realized.

One type of complex systems method is agent-based modeling (ABM). Although a complete discussion of ABM approaches is beyond the scope of this paper and has been published elsewhere [18], a brief overview of the method is provided. Unlike many modeling approaches which seek to identify states of equilibria, ABMs simulate (inter)-actions of heterogeneous, autonomous actors (i.e., “agents”) that may produce non-linear, adaptive, and non-equilibrium dynamics [25]. The model simulates the passage of time in discrete time steps. At each time step, agents update their own internal states based on pre-programmed rules, interactions with other agents, and feedback from their environment. Agents can possess static or varying attributes that influence how behaviors are executed over time. Even simple sets of rules and attributes can result in nonlinear, adaptive, or threshold behavior patterns, which result in the emergence systems-level dynamics [29]. Given that many health and social behaviors are described by these types of processes, ABM have been recognized as crucial for addressing complex public health issues [22], [28]. Of relevance to the work described in this paper, these techniques have been used to understand how substance use is affected by complex and interacting social and environmental factors [15], [30], [31]. Although ABM has been used to investigate infectious disease dynamics for some time [32], [33], [34], few studies have employed these methods to elucidate evolving patterns of HIV transmission, particularly within concurrent sexual and drug-using networks.

**Table 5:** Relationship between adherence to HAART, the per-partnership annualized probability of HIV transmission between serodiscordant agents, and progression to AIDS.

Adherence – *A* (%)	Probability of achieving *A*	Annualized per-partnership probability of HIV transmission			Annual Probability of Progressing to AIDS	Annual Probability of Progressing to AIDS, if IDU
		Syringe sharing	Unprotectedsex betweenmen	Unprotectedheterosexualsex		
Not on HAART	N/A	0.0340	0.0489	0.0100	0.100	0.167
0–29	0.1	0.0340	0.0489	0.0100	0.100	0.167
30–49	0.1	0.0272	0.0391	0.0080	0.082	0.131
50–69	0.1	0.0136	0.0196	0.0040	0.064	0.106
70–89	0.1	0.0068	0.0098	0.0020	0.046	0.083
≥90	0.6	0.0005	0.0008	0.0002	0.010	0.067

Note: per-partnership transmission values derived from a series of Bernoulli distributions, assuming 10 unprotected sexual acts and 5 syringe sharing events (“trials”) per partnership [97], [128], [129], and per-event transmission probabilities of 0.007, 0.005, and 0.001 for syringe sharing, unprotected sex between men, and unprotected heterosexual sex, respectively [123], [124], [125], [126], [127]. We also assume that the relationship between adherence, viral load, and per-event probability of HIV transmission is linear [132], [133]. The relationship between probability of AIDS progression and adherence is also assumed to be linear [63], [143], [144], [145]. Values shown above are multiplied by a factor of four during the first time step following seroconversion to account for increased probability of HIV transmission during early stage infection [130], [131].

### Epidemiology of Substance Use and HIV

Injection drug use is a growing global health concern. Recent estimates suggest that, globally, 16 million people inject drugs, among whom approximately 3 million are HIV positive [35]. Injection drug use is a primary driver of HIV transmission in many settings, including for example parts of Eastern Europe and Central Asia [1].

**Figure 1:**
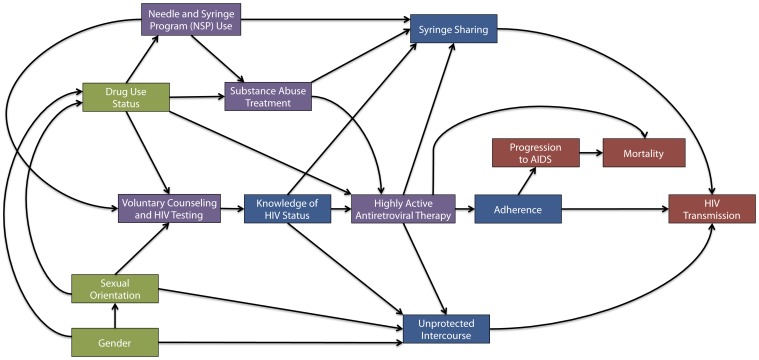
Conceptual framework of the HIV transmission agent-based model. Arrow represents causal effects between two phenomena in the model. For example, NSP use increases an agent’s probability of HIV testing, which is turn can result in both the knowledge of an HIV positive status and initiation of antiretroviral therapy. Green boxes represent agent characteristics that influence individual agent behavior, denoted in blue. Purple boxes represent interventions that influence these behaviors. Red boxes represent model output.

In North America, interventions to reduce injection-related risk behavior have led to significant declines in HIV incidence among IDU populations [36], [37]. Recently however, these reductions have been offset by a continuing rise in HIV prevalence among NIDU [38]. In some settings, including New York City, sexual risk behavior and non-injection drug use have replaced injection-related behaviors as the primary risk factors for HIV infection among IDU, even in the presence of well-established HIV prevention programs [39], [40]. Novel methodologic approaches are required to inform more effective interventions that address evolving time- and place-specific risk factors operating at multiple levels to produce and perpetuate drug-related harms.

Considerable research has demonstrated the effectiveness of a wide array of interventions to decrease HIV transmission among IDU and NIDU [41]. In 2009, the World Health Organization (WHO), the United Nations Office on Drugs and Crime (UNODC), and UNAIDS published a guide for achieving universal access to a combination of evidence-based HIV prevention services for drug users, including NSPs, VCT, opioid substitution therapy (OST) and other forms of substance abuse treatment, and, for HIV positive IDU/NIDU, access to HAART [42]. However, evidence demonstrating the effectiveness of these combination prevention approaches is scarce. Observational studies have suggested that the presence of multiple HIV prevention interventions (e.g., NSPs and OST combined with VCT) can reduce HIV prevalence among some IDU populations to below 10% [43], [44], although in severe epidemics and among particularly disenfranchised sub-groups (e.g., ethnic and sexual minority drug users), these reductions may be more difficult to achieve. A recently published study utilized a compartmental model to suggest that while the provision of NSPs, OST and HAART may result in modest reductions in HIV transmission among IDU, only high-coverage, combination scenarios produce significant population-level benefit [45]. However, it is not clear whether synergistic effects – over and above the additive benefits of implementing multiple interventions – are observed when prevention measures are combined, collocated, or offered in tandem. Furthermore, the precise mechanisms through which combined approaches act to influence systems-level HIV dynamics largely have yet to be elucidated. Accordingly, using complex systems modeling, we sought to assess the hypothetical impact of various combinations of interventions as a means of informing more effective HIV prevention efforts for IDU and NIDU.

**Figure 2:**
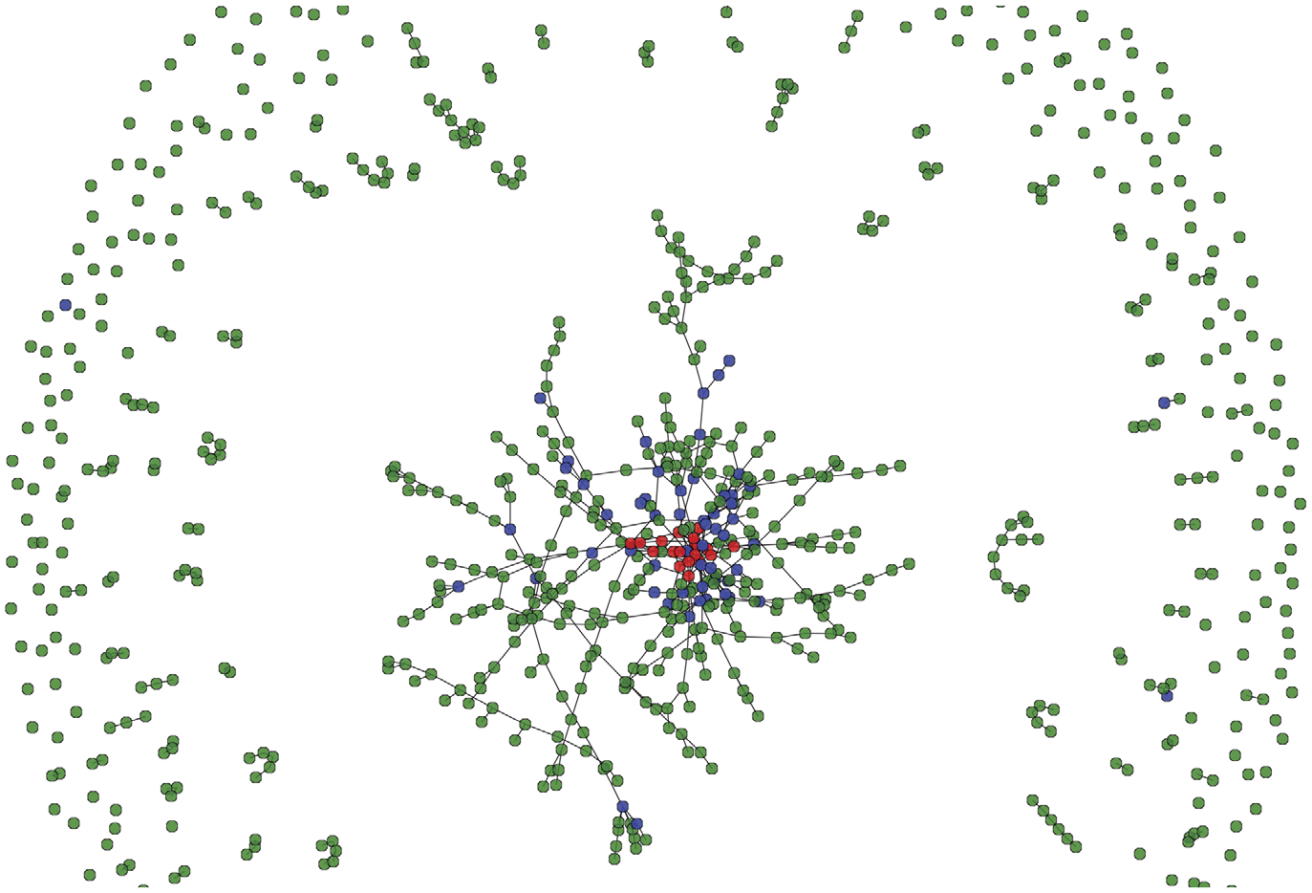
Risk network structure at model initialization of a representative subsample of 1000 agents, stratified by drug use status (IDUs, NIDUs, and NUs are represented by red, blue, and green nodes, respectively). Links indicate sexual activity and/or (if a pair of IDU) injecting event(s) between two agents. In the figures shown above, the average node degree is 1.198.

## Methods

### An Agent-Based HIV Transmission Network Model

We describe an ABM that represents HIV transmission and other transition states (e.g., injection drug use initiation, progression to AIDS) within an artificial society of three categories of agents: IDUs, NIDUs, and non-drug users (NUs). To be consistent with a variety of New York City-based studies of drug users [46], [47], [48], [49], [50], we defined IDUs as agents who are actively injecting drugs (i.e., injected an illicit drug in the past year), and NIDUs as agents who are actively using hard drugs (e.g., crack, heroin, cocaine, methamphetamine) by non-injection routes of consumption (e.g., snorting, smoking). Agents are also stratified by two additional characteristics: sex (female, male), and sexual behavior (men who have sex with men [MSM], heterosexual men [HM], heterosexual women [HW], and women who have sex with women [WSW]). Note that MSM agents include those who engage exclusively in sex with other men and those who have sex with men and women (and analogously for WSW). At each annualized time step, agents interact with other agents and with simulated HIV prevention interventions. A time scale of ten years in annual increments was chosen, as these estimates can be calibrated against empirical data and surveillance statistics.

**Figure 3:**
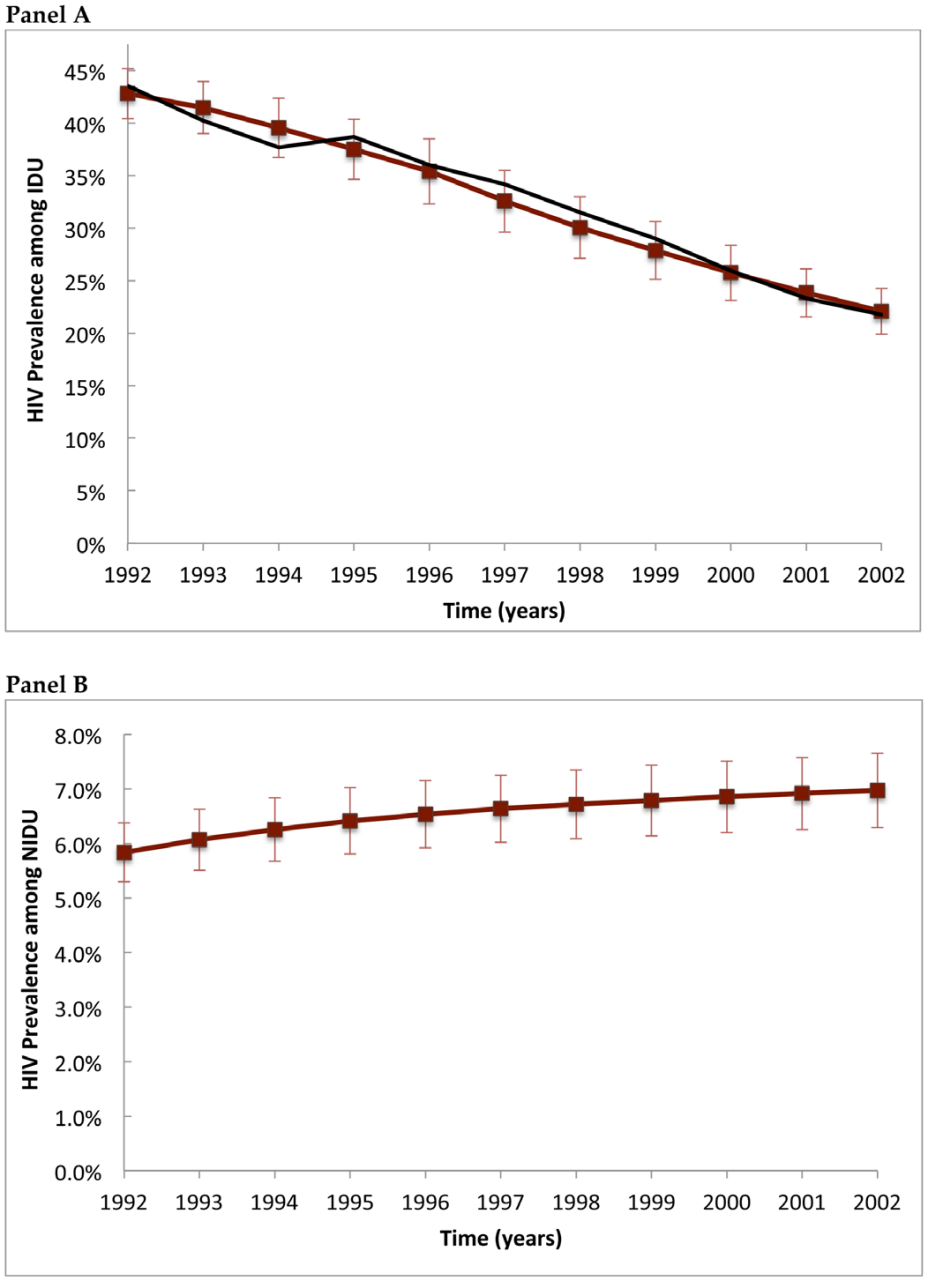
HIV prevalence among IDU (panel A) and NIDU (panel B) obtained from a Monte Carlo simulation of the agent-based model. Black lines indicate empirical trajectories [70]. Error bars represent two standard deviations of the bootstrap estimates; CVAR data for HIV prevalence among NIDU is not available.

We implemented our model using an open-source programming language known as Python™ (version 2.7.2). The simulation consists of an arbitrary population of 100,000 agents, with new agents replacing those who exit the model in a stochastic manner. To initialize the transmission model, agents are constructed and placed in a network space, represented as nodes and links. HIV transmission between serodiscordant agents can occur through unsafe sex, or, if both are IDU, through syringe sharing. The probability of engagement in risk behavior and the conditional likelihood that HIV transmission occurs varies by sex, sexual orientation, and drug use status. The probability of HIV transmission per agent pairing (i.e., across linked nodes) is also dependent on whether agents are engaged in one or more interventions within the model environment. We have focused our initial modeling efforts on four programs (i.e., NSP, substance abuse treatment, VCT, and HAART) which are described by the WHO/UNAIDS/UNODC as “core interventions” within a comprehensive package of HIV-related services for drug users [42]. These interventions have the strongest body of scientific evidence to support their effectiveness [51], and thus constitute the minimally recommended set of interventions to ensure sufficient levels of HIV prevention, treatment and care for IDU populations. Specifically, probability functions stochastically assign each agent in a given time period to: seek substance abuse treatment (a general function that represents all forms of treatment, including OST); if HIV positive initiate HAART, which itself is dependent on accessing VCT; and if an IDU, obtain sterile syringes from NSPs. We hypothesize that the simulated interventions will decrease HIV prevalence, HIV incidence, and AIDS incidence in the agent population through the following pathways of action:

1. Utilization of NSPs will reduce HIV transmission through the provision of sterile injecting equipment [52]. NSP use will also result in an increased uptake of other HIV prevention services, namely substance abuse treatment [53], [54], [55], [56].
2. HIV-infected agents who access VCT can initiate HAART. Studies have demonstrated that early initiation of HAART can effectively eliminate HIV transmission between serodiscordant partners by suppressing viral load [4], [57], [58], [59]. HIV positive agents who initiate HAART will also be less likely to engage in sexual risk behavior and are less likely to progress to AIDS [60], [61], [62], [63].
3. Drug-using agents can enroll in substance abuse treatment, which increases the probability of initiating HAART and reduces engagement in syringe sharing [64], [65], [66].

In order for ABM simulation systems to produce reasonable values for unobserved variables (i.e., distributions of risk behaviors among subgroups of users), model parameterization and calibration should be based whenever possible on empiric data [67]. In addition to previously published estimates, this model has been calibrated against data collected as part of a study known as the Community Vulnerability and Responses to Drug User-Related HIV/AIDS (CVAR). As described previously [68], [69], [70], robust methods were used to estimate annual IDU prevalence, HIV prevalence, and AIDS incidence between 1992 and 2002 within the 96 largest metropolitan statistical areas (MSAs) in the United States. Although one objective of future modeling work is to replicate historical HIV prevalence and predict HIV dynamics in any given MSA, the model presented here has been parameterized and calibrated using HIV prevalence estimates for the New York MSA (population 11.7 million in 2010).

**Figure 4:**
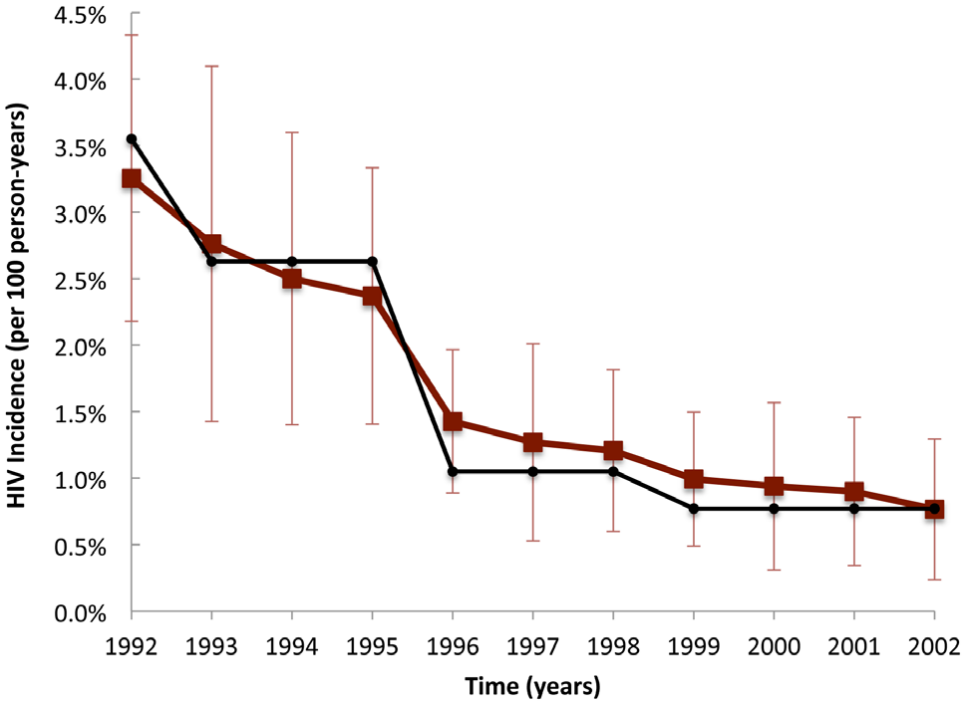
HIV incidence among IDU obtained from a Monte Carlo simulation of the agent-based model. Data shown in black represent empirically observed estimates HIV incidence among IDU in New York City [36]. Error bars represent two standard deviations of the bootstrapped estimates.

### Network Structure: Modeling Risk

At model initialization, initial conditions are set such that the agent population represents a population-based sex and drug-using network of individuals living in the New York MSA in 1992. When the model is initialized, sexual orientation and drug use status are attributed randomly to agents, such that 6.0% of male agents are MSM [71], [72], and 5.0% of female agents are WSW [73]. We consider 1.9% of the total 1992 population to be IDUs, 70% of whom are male [68], [69], while 6.4% of the initial population is an NIDU [74], 60% of whom are male [75]. Additionally, MSM and WSW are overrepresented among drug-using agents [76], [77], [78], [79], [80]. Conditional distributions of agent characteristics are shown in Table 1.

**Figure 5:**
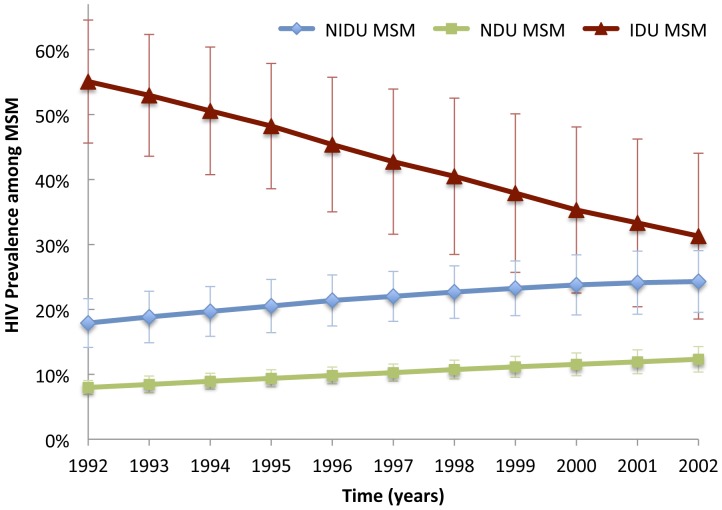
HIV prevalence among MSM agents obtained from a Monte Carlo simulation of the agent-based model, stratified by drug user status. Error bars represent two standard deviations of the bootstrap estimates. Abbreviations: MSM = men who have sex with men; NU = non-drug user; NIDU = non-injection drug user; IDU = injection drug user.

At model initialization and at each time step thereafter, a network is constructed such that each index agent interacts (i.e., has sex or injects with) *k* others in the agent population, where *k* is greater than or equal to zero. Agents are only connected to other agents with whom sexual- or injecting-related risk behavior can occur (i.e., we model a risk network as opposed to a social network). For example, two heterosexual male NUs cannot be connected to each other, whereas two heterosexual male IDUs can be linked since syringe sharing between the pair can occur. For non-IDU MSM agents, we incorporate assortative mixing (i.e., favoring links between nodes with similar characteristics) such that 90% interact exclusively with other MSM [81], [82], while the remaining 10% are connected randomly to other agents with whom they can have sex (i.e., other MSM, WSW, and heterosexual females). Given studies suggesting that a high proportion of IDU-MSM engage in sexual activity with women [83], [84], [85], we assume only 50% of this group interacts exclusively with other MSM. For WSW agents, 50% are sexually connected to WSW exclusively [73]; the remaining 50% are assigned random agents with whom they can have sex (i.e., other WSW, MSM, and heterosexual males). The value of *k* for each agent varies per time step, and is specified by a random variable sampled from a probability distribution function for the following five categories of agents: IDU, NIDU, NU MSM, NU WSW, and the general population (i.e., non-IDU, non-NIDU, non-MSM, non-WSW agents). Although alternative degree distributions (e.g., negative binomial, discrete Pareto) can be implemented and will be explored in future research, we have defined *k* as a random variable sampled from a series of Poisson distribution functions. This distribution assumes partners are acquired at a fixed homogeneous rate (λ) over time [86]. Although real-world social and sexual networks are often highly skewed and can deviate substantially from the Poisson model, the node degree distribution of the widely used Erdős-Rényi random network follows a Poisson distribution and thus will be used as an approximation [87]. For IDU, we assume a mean (λ) of five annual injecting and/or sexual contacts [88], [89], [90]. For links between IDU through which both sexual and injecting behavior can occur, we define a random probability of engaging in sex only at 0.20, a probability of injecting only at 0.60, and a probability of doing both at 0.20 [88], [89], [90]. We incorporate assortative mixing for IDU, such that, at any given time step, IDUs are four-fold more likely to establish a connection with another IDU compared to other agent types [88], [89], [90]. For agents who are NIDU, we let λ = 3 [91], [92], [93]. We also incorporate assortative mixing for NIDU, such that each NIDU agent has a 0.12 probability of being connected to at least one IDU per time step [94], [95]. Additionally, each NIDU has a 0.50 probability of being connected to at least one other NIDU during each time step [96]. These attachment probabilities (and those governing mixing for MSM and WSW) are applied at each time step such that the assortative mixing patterns specified at model initiation are retained. For non drug-using MSM and WSW agents, we assume a Poisson distribution with λ = 1.5 [73], [82], [97]. For all other agents, the number of contacts per time step is sampled from a Poisson distribution with a mean (λ) of 1.0, which corresponds to empirically observed distributions of sexual partnerships in the general US population [98], [99].

We note that randomly re-assigning contact values (*k*) at each time step would overestimate sexual and injecting partnership turnover and underestimate partnership duration. Therefore, we incorporate a counting process, whereby agents are added or removed from index agent *i*’s network according to the random number drawn at each time step. For example, assume *k* = 7 for agent *i* at *t* = 0. If the random variable sampled from the Poisson distribution at *t* = 1 produces a value of *k* = 9, agent *i* adds two new agents to the seven she/he is already connected to. If at *t* = 2 the random variable produces *k* = 5, agent *i* loses 4 links from its network, chosen stochastically. The simulation proceeds sequentially through the list of agents such that the population’s degree distribution is updated in an iterative manner. For example, if agent *i* loses agent *j* from its network, and agent *j*’s degree distribution is specified to remain constant at that time step, a new link will be formed between *j* and a third agent *k*, who is chosen from the remaining agents who have not yet been updated at that time step. This algorithm produces estimates for partnership duration and turnover that are similar to those observed in a network study of IDU in Bushwick, Brooklyn [47], a population-based study of MSM in New York City [82], and other nationally representative sexual behavior studies [73], [98], [100], [101].

### Agent Risk Behavior

Agents engage in two types of HIV risk behavior: unprotected intercourse, and, if both agents are IDUs, syringe sharing. Unprotected intercourse is defined as the annual probability of less than 100% correct and consistent condom use between two agents. The probability that two agents engage in unprotected intercourse varies by drug use status and sex/sexual orientation, the values of which are shown in Tables 2, 3, 4. These values have been parameterized by a nationally representative survey of sexual behavior among US adults [101], and population-specific cohort studies for IDU, NIDU, WSW, and MSM [78], [80], [83], [102], [103], [104], [105], [106], [107], [108], [109], [110]. Since studies have shown that persons who are aware of their HIV positive status are less likely to engage in sexual risk behavior [60], [111], [112], [113], we assume the probability that a pair of agents engages in sexual risk will be 50% lower if one or both has tested positive (i.e., accesses VCT). While evidence suggesting risk behavior change following HIV diagnosis among MSM is mixed and evolving [114], several reviews have suggested that testing positive has a negligible impact on sexual risk behavior in this population [61], [115]. Therefore, we assume that for MSM, the probability of engaging in sexual risk behavior is not dependent on VCT.

IDU agents practice sexual risk behavior and can also share used syringes with other IDUs. Several studies of New York City IDU have shown that the annual probability of consistent NSP use (i.e., no syringe sharing) among IDUs in 1992–1994 was approximately 0.65 [47], [116]. Due to an expansion of NSPs and other policy and programmatic changes in the mid-1990’s the probability of NSP use in later years increased to 0.80 [44]. To match these two time periods [44], we model a step function such that *P*(NSP use)*_t_*_ = 1992–1994_ = 0.65 and *P*(NSP use)*_t_*_≥1995_ = 0.80.

Agents who are IDU can also enter substance abuse treatment. We note that NIDU do not interact with substance abuse treatment, an assumption that will be relaxed in future iterations of the model. At model initialization, we stochastically assign 9% of IDUs to be in substance abuse treatment [117]. Based on previously published estimates, we set the annual probability of entering substance abuse treatment for IDU to be 0.09 [53], [118]. Once an IDU enters treatment during time step *t*, the probability of remaining in treatment at *t*+1 is 0.5 [53], [119]. Once a user relapses, the probability of (re)-entering treatment doubles for all future time points, based on literature demonstrating that prior treatment exposure is a strong predictor of re-entering treatment [53], [118], [120]. A recently published Cochrane review has demonstrated that in-treatment IDUs are approximately 50% less likely to share syringes than out-of-treatment IDUs [66]. Therefore, in our model, we will assume that enrollment in substance abuse treatment increases the likelihood of NSP use (i.e., decreases the probability that a pair of IDU share syringes), such that: *P*(NSP use*_t_* |treatment*_t_* )*_t_*_ = 1992–1994_ = 0.85 and *P*(NSP use*_t_* |treatment*_t_*)*_t_*_≥1995_ = 0.90. We assume no change in sexual behavior while IDUs are in treatment [66], [121]. We also assume that the networks of IDUs in treatment are the same as IDUs out of treatment. Finally, given studies demonstrating that consistent NSP utilization doubles the likelihood of accessing drug treatment [53], [55], [56], [122], we assume the probability of entering treatment during time *t* given NSP use at time *t* is twice the value for IDUs who do not use an NSP. In this manner, the likelihood of engagement in either or both of these interventions is explicitly linked and interdependent.

Agents access VCT with probabilities shown in Tables 2, 3, 4. Note that for an IDU who utilizes an NSP at time *t*, the probability of VCT at time *t* increases by a factor of 1.25 [54]. An agent who tests HIV positive after 1996 can initiate HAART. The likelihood of initiating therapy varies by drug use status; for IDU, being enrolled in substance abuse treatment increases the probability of commencing HAART [65].

### HIV Transmission and Progression

The probability that an HIV negative agent acquires infection from a serodiscordant partner is derived from empirical estimates of per-act HIV transmission: 0.007 for syringe sharing [123], 0.005 for unprotected intercourse between men [124], [125], [126], and 0.001 for unprotected heterosexual intercourse [125], [127]. To obtain the per-partner probability of HIV transmission between serodiscordant agents, we model a series of Bernoulli distributions, *p_partner_* = 1– (1– *p_act_*)*^n^*, where *p_partner_* is the annualized per partnership risk of HIV transmission, *p_act_* is the per act “transmission event” probability described above, and *n* is the number of “trials” per partnership per time step. We assume that, if two agents practice a risk behavior during a time step, the pair engages in a total of 10 unprotected sexual acts (“trials”) and/or, if IDUs, 5 syringe sharing events per annum [97], [128], [129]. The resulting annualized per partnership risks of HIV transmission are shown in Table 5. In order to accommodate increased transmission risk during early stage HIV infection and to be consistent with studies of per-coital rates of HIV transmission by stage of infection [130], [131], we multiplied these probabilities by a factor of four during the first time step following seroconversion.

In order to examine the potential influence of HAART on disease acquisition at the individual and population level, we model the relationship between adherence, viral load, and per-event HIV transmission. A landmark study by Bangsberg *et al* demonstrated that the relationship between adherence and log_10_(viral load) is approximately linear and highly correlated (*r = *0.8) [132]. Furthermore, an important study by Quinn and colleagues and a recently published meta-analysis allow us to model the relationship between viral load and probability of HIV transmission between serodiscordant partners [133], [134]_ENREF_80. Once an agent initiates HAART, we stochastically assign an adherence value that we assume does not change over the course of therapy. Higher values of adherence reduce the per-event probability of transmission (and thus the per-partner transmission probability) as shown in Table 5. Based on prior literature and a systematic review [135], [136], [137], [138], we assume 60% of agents achieve ≥90% adherence upon initiating HAART (all other adherence values are assumed to be equally likely). Although some studies have shown that IDU tend to be less adherent than non-IDU [135], [137], a recent meta-analysis suggested that this may not be the case [138]. Therefore, we assume no relationship between drug use and adherence.

Prospective cohort studies have shown that approximately 45% of IDU who initiate HAART discontinue therapy after 1 year [65], [139], [140], [141]. In contrast, approximately 35% of NIDU and NU who initiate HAART discontinue therapy after 1 year [141], [142]; therefore, we assume *P*(HAART discontinuation| IDU, NIDU) = 0.45 and *P*(HAART discontinuation| NU, NIDU) = 0.35. Agents who discontinue therapy during time *t = i* can re-initiate therapy at any time *t* > *i*.

HIV positive agents progress to AIDS in a stochastic manner. For HIV positive agents who are not on HAART, the annual probability of AIDS progression is 0.10 [143], [144], while those on HAART with ≥90% adherence progress to AIDS at an annual probability equal to 0.01 [63]. We also model the relationship between <90% adherence on the probability of AIDS progression; empiric values are shown in Table 5 and derived from previously published data [145]. As shown in Table 5, IDU have an increased likelihood of progressing to AIDS [144].

### Rates of Drug Use Transitions and Mortality

At each time step, an NU who interacts with an NIDU can transition to being an NIDU (and vice versa), and an NIDU who meets an IDU can transition to being an IDU. We assume the probability that an NU transitions to becoming an NIDU (and vice versa) is 0.001 per time step. If an NIDU has contact with an IDU during time *t*, the probability that the agent will transition to an injecting state is 0.018. These values were estimated inductively from the calibration procedure (see below) that sought to reproduce the observed empirical prevalence of drug use (from CVAR data) over the lifetime of the model. In addition to transitions in drug use that are dependent on social contact, IDU and NIDU have a 0.017 probability of spontaneous drug use cessation per time step [146]. Once IDU cease injecting, they take on the behaviors and networks of NIDUs, and when NIDUs cease drug use, they take on the properties of NUs.

At each time step, agents exit the model according to a probability that is dependent upon three factors: HIV status, drug use status, and HIV treatment status. These probabilities are derived population-based mortality studies and other relevant prospective cohorts and are shown in Tables 2, 3, 4.

### Model Calibration

To calibrate the ABM, we used an iterative indirect approach that has been described in detail previously [147]. Briefly, we first identified which real-world phenomena we were interested in reproducing (i.e., drug use prevalence, HIV prevalence/incidence), and subsequently developed a conceptual framework to guide the selection of processes and behaviors that would be modeled in the agent-based environment (see Figure 1). As a second step, we sought to construct a model that reflected known empirical and experimental evidence about these behaviors.

As a third step, we calibrated the model through an iterative process by which model output was compared to CVAR data and other empirical estimates. Model refinement was conducted by comparing model output with these datasets, adjusting key parameters (e.g., probability of sexual and injection risk behavior, interaction with various interventions) to minimize differences, and running a revised set of simulations. The central tenet of this approach is that the artificial generation of patterns in modeled output that reflects empirically observed phenomena helps to affirm (but does not necessarily guarantee) the validity of the ABM, and has been used successfully in a number of recent studies [148], [149]. This process permitted the identification of parameter spaces and network structures that did not adequately reproduce the empirical data; for example, network structures without assortative mixing significantly underestimated HIV prevalence and incidence in some subpopulations (data not shown). We continued refining the model parameters and initial conditions until the output from simulations qualitatively matched the empirical estimates.

As a final step, we employed Monte Carlo techniques to examine the degree of variation in model outputs arising from the many processes and behaviors that are stochastic. A simulation of 100,000 agents over 11 time steps was repeated 1,000 times. To run the simulation, we used a Beowulf computing cluster consisting of 6 compute nodes and 1 head node, each with two quad-core Intel™ CPUs and between 8 and 24 GB of RAM. In the figures below, we show the mean and two standard deviations for each estimate from the sampled distributions.

## Results

### The Risk Network

A representative network structure of a random subsample of 1,000 agents at model initialization is shown in Figure 2. The findings are qualitatively similar to empirical sexual and injecting network studies in New York City and Colorado Springs [85], [150]. Namely, the ABM risk network consists of a very large central “core” component, with a cluster of IDU at its center. Furthermore, we observed both cyclic (multiple pathways between network members) and dendritic (linear chains of connections between nodes) microstructures, which are common characteristics of sexual and drug-injecting networks [151]. Finally, we note the presence of network members who appear to act as “bridges” between smaller components and the central core. Although beyond the scope of this paper, a formal network analysis will be conducted to confirm quantitatively whether the modeled network accurately reflects real-world sexual and injecting network topologies.

### HIV Trajectories among Injecting and Non-injecting Drug Users

The model generated injecting and non-injecting drug user prevalence approximately similar to that reported by CVAR [68], including a steady decline in the proportion of agents who are IDU (data not shown). In figures 3, 4 and 5, we show the model predictions for HIV prevalence and incidence among key sub-populations of interest, including for example an approximately linear decrease in HIV prevalence among IDU observed between 1992 and 2002 [70]. The estimated prevalence of HIV among NIDU (i.e., 7% at 2002) is consistent with a previously published estimate [95], but lower than one other study that found an HIV prevalence of 12% among never injectors [38]. Although HIV incidence data were not estimated by CVAR, the ABM generated HIV incidence trajectories that are similar to previously published estimates (Figure 4) [36]. Finally, the model produced estimates of AIDS incidence that are consistent with unpublished CVAR data (not shown).

### HIV Trajectories among MSM

In addition to modeling HIV prevalence and incidence among IDU/NIDU, the population-based nature of the ABM also permitted an examination of HIV trajectories in other subgroups, including MSM. In Figure 5, we demonstrate that trends in HIV prevalence among MSM vary substantially by drug use status. While HIV prevalence among MSM-IDU declines over the lifetime of the model, infection in MSM-NIDU and MSM-NU increases significantly. These findings are broadly consistent with findings from the New York site of the CDC National HIV Behavioral Surveillance system and other studies, demonstrating declining HIV prevalence among MSM IDU between 1990 and 1999 [102], and continuing propagation among NIDU and NU MSM over the past two decades [152], [153]. For example, HIV prevalence in a probability sample of New York City NIDU and NU MSM in 1997 was 12 and 24 percent, respectively [81], which are similar to those generated by the ABM (i.e., 14 and 27 percent).

## Discussion

Through the development and calibration of an agent-based model, we were able to closely approximate trends in the HIV epidemic observed historically in New York City. In contrast to deterministic compartmental models such as SIR models (see for example [45], [154]), our model allows for the monitoring of individual agent behavior and HIV disease progression among infected persons. Additionally, the agent-based model allows for greater heterogeneity in the simulated population (e.g., gender, sexual orientation, drug use status, HIV disease status) than most existing network models, including for example several exponential random graph network models of HIV transmission [155], [156]. Finally, the ABM allows for an examination of how interdependence and feedback between simulated sets of prevention interventions influence population-level HIV transmission dynamics over time. Although future work is required to confirm these exploratory results, explicit specification of non-independent agent-intervention interactions reproduces estimates that approximate empirically observed phenomena.

Although combination HIV prevention has garnered much recent attention and will likely be a central component of successful worldwide HIV strategies over the coming decade [8], [13], [14], several authors have noted the absence of data to inform how best to combine available evidence-based interventions and how to optimize their effectiveness [45], [157]. We have demonstrated the capacity for complex systems approaches to overcome many of the methodologic challenges inherent in observational studies (e.g., cost, difficulty capturing non-linear adaptive dynamics), and the potential for these methods to model “real-world” policy scenarios. In future work, we will use the calibrated ABM described herein to formally model the hypothesis that interventions operating in a coordinated and comprehensive manner will substantially reduce (and potentially eliminate) HIV transmission at the population level.

In addition to informing future studies that seek to investigate combination HIV prevention approaches, these results also illustrate the utility and relevance of complex systems approaches within social epidemiology and HIV prevention science. Although agent-based models are increasingly common in the field [31], [149], [158], the methods are not without challenges and skepticism [18], [159]. For this reason, a key objective of this paper was to provide readers with a detailed protocol for model development and calibration that can be duplicated and improved upon. Although standard protocols for reporting complex systems methods have been published in other fields including ecology [160], acceptable standards and conventions for reporting the results of epidemiologic ABMs require further development and implementation.

The construction and calibration of this ABM was not absent of challenges, and the study has a number of important limitations that bear mentioning. First, the replication of historical patterns does not necessarily imply that model assumptions and processes have been correctly specified [149]. To further support model validity, findings derived from ABM simulations should be robust to changes in critical assumptions regarding network topology and agent behavior, parameter values, and initial conditions [161]. This will be the primary focus of future work. Second, although we have based model parameters on existing data wherever possible, for some features (e.g., drug use transitions) empirical data were not available. In order to realize the full potential of the complex systems methods, model development should proceed in tandem with empirical data collection, such that the two scientific processes inform each other. Third, ABM behavior can be heavily dependent on system size [161]. Although we conducted simulations over a range of model sizes and did not identify effects of system size on resulting behaviors, we cannot preclude the possibility that a relationship between model size and system behavior exists. Fourth, although we chose a relatively coarse timescale (i.e., annual time steps) to reduce computational resource requirements and to model HIV trajectories over a long time period (e.g., 11 years for model calibration), the model likely underestimates the effect of short-term behavioral dynamics, including for example partner concurrency. A time domain of finer resolution might have allowed for the modeling of these short-term effects that are currently not considered and will be the focus of future work.

We conclude by emphasizing that the model has substantial room for continued refinement and validation. For example, the network topology is relatively basic, and important aspects of real-world networks (e.g., social norms and the network and individual properties that shape who forms a relationship with whom) were not considered. For example, while some empirical research suggests that IDUs enrolled in substance abuse treatment have fewer drug-using network members and are more likely be to located at the periphery of the network [47], [162], we assumed in-treatment IDUs have the same network characteristics as IDUs out-of-treatment. Furthermore, although we have explicitly incorporated sex and sexual orientation in the model, other sociodemographic characteristics (e.g., ethnicity) are not included. Finally, we must continue to test model robustness, particularly in terms of the ABM’s sensitivity to changes in parameters, network topologies, and other key assumptions.

ABMs constitute a novel analytic approach that complements other scientific modes of inquiry, offering key insights into the properties, dynamics, and evolution of complex systems. Although not without challenges, these methods hold much promise for improving our understanding of HIV risk, drug use, and other health behaviors as they operate within adaptive environments and complex social systems.
